# 538 Electrospun Mafenide Acetate Wound Dressing for Treatment of Invasive Burn Wound Infection

**DOI:** 10.1093/jbcr/iraf019.167

**Published:** 2025-04-01

**Authors:** Mia Mae Kiamco, Eliza Sebastian, Andrea Fourcaudot, David Silliman, S L Rajasekhar Karna, Ping Chen, Joseph Wolf, Kai Leung

**Affiliations:** US Army Institute of Surgical Research; US Army Institute of Surgical Research; US Army Institute of Surgical Research; US Army Institute of Surgical Research; US Army Institute of Surgical Research; US Army Institute of Surgical Research; US Army Institute of Surgical Research; US Army Institute of Surgical Research

## Abstract

**Introduction:**

Burn injuries have occurred in 5–15% of combat casualties during recent conflicts. More burn casualties with larger wound size and increased severity are anticipated in the future large-scale combat operation (LSCO). Burn wound infections are the leading cause of death in burn patients and are predictor of mortality in acute burn patients. Mafenide acetate (MA) cream, readily penetrates the burn eschar and is used on burn patients from the early 70’s onward for reducing mortality caused by Pseudomonas aeruginosa invasive burn wound infections. MA from the cream readily enters the wound, attaining peak levels of 2- to 5-fold above the minimal inhibitory concentration (MIC) for P. aeruginosa at 1–2 hrs, but rapidly declines to subinhibitory concentrations by 8–10 hrs. To sustain the treatment effect, repeated applications (every 6 hrs) of MA cream are required, a practice that is non-conducive in LSCO with mass casualties. Thus, we propose an electrospun burn wound dressing that sustainedly releases MA at therapeutic concentrations into burn wounds for ≥ 24 hrs.

**Methods:**

Electrospun nanofibers of MA were fabricated using electrospinner. Fiber morphology was imaged using Scanning Electron Microscope (SEM). In vitro release and burned rat skin permeation tests were performed using a Franz cell system. A modified Walker-Mason rat model of deep partial-thickness dorsal scald burn was used to determine concentrations of MA in burned skins after 24 hrs. All rats were anesthetized with isoflurane and analgesia with BuprenorphineSR was provided prior to and post all procedures. Drug activity was determined using a modified AATCC 100 test method against six commonly found burn wound pathogens.

**Results:**

The SEM images of electrospun MA fabric (dressing) showed the presence of smooth and uniformly sized fibers. The wetted electrospun MA fabric demonstrated sustained release of MA up to 24 hrs as shown by the in vitro release assay; around 10-20 mg/cm²/hr of MA was released from the MA fabric whereas around 1 mg/cm²/hr was released from the MA cream. Additionally, electrospun MA fabric released approximately 5× more MA than MA cream into burned rat skins in vitro. Furthermore, electrospun MA fabric delivered 3-8 mg MA per gram of burned skin (at and above MIC for P. aeruginosa) in rats. Lastly, electrospun MA fabric showed a potent antimicrobial activity, causing a 7-9 log reduction of bacterial counts against the test burn wound pathogens.

**Conclusions:**

The electrospun MA fabric dressing exhibited sustained release of MA in vitro and in vivo and demonstrated potent antimicrobial activity against burn wound pathogens in vitro.

**Applicability of Research to Practice:**

To develop an effective burn wound dressing that targets invasive burn wound infection and reduces patient care burden.

**Funding for the Study:**

This work was supported by the Intramural Program.

